# MODY-like diabetes associated with an apparently balanced translocation: possible involvement of *MPP7 *gene and cell polarity in the pathogenesis of diabetes

**DOI:** 10.1186/1755-8166-2-5

**Published:** 2009-02-13

**Authors:** Elizabeth J Bhoj, Stefano Romeo, Marco G Baroni, Guy Bartov, Roger A Schultz, Andrew R Zinn

**Affiliations:** 1 McDermott Center for Human Growth and Development, The University of Texas Southwestern Medical Center, Dallas, Texas 75390, USA; 2 Department of Medical Sciences, Endocrinology, University of Cagliari, Cagliari, Italy; 3 Department of Pathology, The University of Texas Southwestern Medical Center, Dallas, Texas 75390, USA; 4 Department of Internal Medicine, The University of Texas Southwestern Medical Center, Dallas, Texas 75390, USA; 5 Signature Genomic Laboratories, LLC, Spokane, WA, USA

## Abstract

**Background:**

Characterization of disease-associated balanced translocations has led to the discovery of genes responsible for many disorders, including syndromes that include various forms of diabetes mellitus. We studied a man with unexplained maturity onset diabetes of the young (MODY)-like diabetes and an apparently balanced translocation [46,XY,t(7;10)(q22;p12)] and sought to identify a novel diabetes locus by characterizing the translocation breakpoints.

**Results:**

Mutations in coding exons and splice sites of known MODY genes were first ruled out by PCR amplification and DNA sequencing. Fluorescent in situ hybridization (FISH) studies demonstrated that the translocation did not disrupt two known diabetes-related genes on 10p12. The translocation breakpoints were further mapped to high resolution using FISH and somatic cell hybrids and the junctions PCR-amplified and sequenced. The translocation did not disrupt any annotated transcription unit. However, the chromosome 10 breakpoint was 220 kilobases 5' to the *Membrane Protein, Palmitoylated 7 *(*MPP7*) gene, which encodes a protein required for proper cell polarity. This biological function is shared by *HNF4A*, a known MODY gene. Databases show *MPP7 *is highly expressed in mouse pancreas and is expressed in human islets. The translocation did not appear to alter lymphoblastoid expression of *MPP7 *or other genes near the breakpoints.

**Conclusion:**

The balanced translocation and MODY-like diabetes in the proband could be coincidental. Alternatively, the translocation may cause islet cell dysfunction by altering *MPP7 *expression in a subtle or tissue-specific fashion. The potential roles of *MPP7 *mutations in diabetes and perturbed islet cell polarity in insulin secretion warrant further study.

## Background

Although common diabetes mellitus is polygenic, there are also rare Mendelian forms of the disease. Maturity-onset diabetes of the young (MODY) is a collection of uncommon monogenic insulin-secretion pathologies. It was first described in 1960 in young lean patients who had only mild diabetes, with little progression after years of follow up [1]. Clinical criteria for MODY include autosomal dominant inheritance, onset before age 30, correction of fasting hyperglycemia without insulin for at least two years post-diagnosis, and absence of ketosis. The estimated contribution to the total diabetic population ranges from 2–5% [2].

It was hypothesized that the genes that cause MODY also contribute to the genetic susceptibility towards common type 1 and 2 diabetes. However, multiple studies have failed to demonstrate such a connection conclusively beyond a few individual examples, and large-scale non-biased genome-wide linkage and association studies have identified several alternate candidate genes for type 1 and 2 diabetes not implicated in MODY [3,4]. However, the identification of MODY genes has provided important insights into molecular mechanisms of glucose homeostasis. There are six well-established MODY genes: Hepatocyte Nuclear Factor 4 alpha (*HNF4A*), Glucokinase (*GCK*), Hepatocyte Nuclear Factor 1 alpha gene (*HNF1A*), Insulin Promoter Factor 1 (*IPF1*), Hepatocyte nuclear factor 1 beta (*HNF1B*), and Neurogenic Differentiation 1 (*NEUROD1*). In each case the mechanism of dominance is thought to be haploinsufficiency [5,6]. There is also a population of MODY patients who have no identifiable mutations in any of the known causative genes; they are sometimes called MODY-X and may harbor mutations in yet-to-be described MODY genes. The proportion of MODY patients with MODY-X is variable among ethnicities, ranging from 20% of Caucasians to 80% of Japanese [5].

Balanced translocations have been used to localize genes responsible for a variety of conditions. Translocations are likely to mediate disease processes by disrupting expression of genes in the vicinity of the breakpoints. The first disease whose genetic cause was identified by mapping of a balanced chromosomal translocation breakpoint was chronic granulomatous disease [7]. Subsequently, genes responsible for a variety of conditions, such as obesity, cleft palate, blepharophimosis syndrome, DiGeorge syndrome, Duchene muscular dystrophy, and congenital cataracts, have been identified using this strategy [8-13].

A number of translocations have been associated with diabetes. In one family a balanced translocation between chromosomes 3 and 20, involving the promoter of *HNF4A*, co-segregated with MODY [14]. In another interesting case, a patient presented with an unbalanced translocation resulted in monosomy of part of Xq and trisomy of a portion of 10p. She demonstrated a MODY-like phenotype and primary amenorrhea at age 16. The authors suggest that the Xq monosomy is responsible for the diabetic phenotype since other patients with X chromosome deletions have demonstrated an increased incidence of diabetes [15]. In another family with a balanced translocation and type 2 diabetes, the candidate gene *inositol hexaphosphate kinase 1 *(*IHPK1*) was identified by mapping the translocation breakpoints [16]. However, the authors were unable to find any *IHPK1 *mutations in 405 other diabetic patients screened. Mutations in the *ALMS1 *gene responsible for Alstrom syndrome, a recessive disease characterized by blindness, sensorineural hearing loss, early onset, and type 2 diabetes mellitus, were identified from study of a subject who was a compound heterozygote for an intragenic mutation and a balanced translocation that disrupted the gene [17]. Recently a woman with intrauterine growth retardation, short stature, lactation failure, and insulin resistance with altered fat distribution was found to have a balanced translocation that disrupted the paternally-derived *Insulin-like Growth Factor 2 *(*IGF2*) gene [18]. Her daughter inherited the translocation but was clinically unaffected, consistent with known *IGF2 *maternal imprinting.

We report a subject with MODY-like diabetes and an apparently balanced translocation [46,XY,t(7;10)(q22;p12)]. We hypothesized that the translocation disrupted a diabetes gene. To test this hypothesis, we mapped both translocation breakpoints to nucleotide resolution and studied the expression of candidate genes near the breakpoints. We identified a novel candidate diabetes gene, *MPP7*, near the breakpoint on chromosome 10.

## Methods

### Clinical report

This study was approved by the Institutional Review Board at UT Southwestern Medical School. Informed consent was obtained from participants. The proband was an Italian man found at age 32 to have incidental hyperglycemia (serum glucose 220 mg/dL) during an evaluation for a minor gastrointestinal ailment. After workup for hyperglycemia, he was given a diagnosis of probable MODY because of his relatively low body mass index (BMI) (26.8 kg/m^2^), lack of Glutamic Acid Decarboxylase 65 (GAD65) antibodies, and clinical evidence of defective glucose-stimulated insulin secretion and normal insulin sensitivity. Over the subsequent eight years he was treated with diet alone, and based on homeostatic model assessment [19,20] calculated with the updated computerized model , his estimated beta cell insulin secretory function secretion decreased by only 8%. His family history is notable for type 2 diabetes mellitus in his mother, associated with obesity (BMI 30.8 kg/m^2^), hypertension, hypertriglyceridemia, and macrovascular complications. Her beta cell insulin secretory function decreased by 50% over the nine years since her diagnosis at 55 years of age. The proband's birth weight was not available.

**Figure 1:**
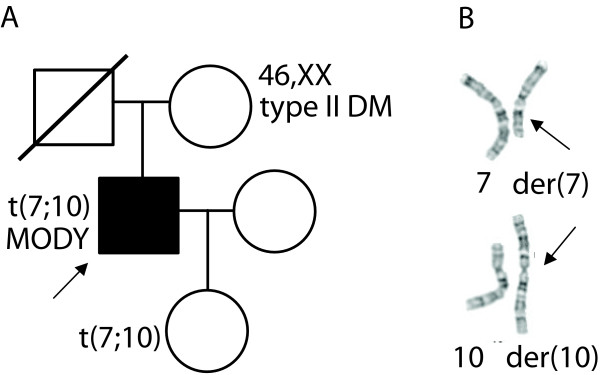
**A. Pedigree of proband with MODY-like diabetes and a balanced 7;10 translocation**. B. Partial G-banded karyotype of proband showing normal and derivative chromosomes 7 and 10. Arrows indicate breakpoints.

The proband's apparently balanced translocation was discovered after a prenatal karyotype for advanced maternal age showed that his daughter carries the same translocation, 46,XY,t(7;10)(q22;p12) (Fig. 1). The proband's mother had a normal karyotype, and his deceased father's chromosomal status is unknown. At the time of the study the daughter was six years old and in good health, with no significant past medical history. Her birth weight was not available, and her parents did not permit her to undergo any clinical or research testing.

### Genomic sequencing

Genomic DNA was purified from peripheral blood leukocytes by standard methods [21]. Primers were designed to PCR-amplify coding exons of *HNF4A*, *GCK*, *HNF1A*, *IPF1*, and *NEUROD1 *using Primer3 [22]. Products were treated with ExoSAP (USB Corp, Cleveland, OH) and sequenced with the same primers used for PCR. Sequencing data were analyzed using Seqman (DNAStar, Madison, WI).

### Oligonucleotide Array Comparitive Genomic Hybridization (CGH)

Lymphoblastoid cells were immortalized by standard techniques. Genomic DNA was purified as described above and submitted to Nimblegen Systems Inc. (Madison, WI) for whole genome array CGH using an array containing ~385,000 probes (Cat. No. B4366-00-01), with pooled normal human male reference DNA (Promega, Madison, WI). CGH segmentation data were compared with the Database of Genomic Variants [23] to determine if there were any copy number changes not previously described in normal individuals.

### Fluorescence in situ hybridization (FISH)

Metaphase chromosomes from either PHA-stimulated whole blood lymphocytes or immortalized lymphoblasts were used for fluorescent in situ hybridization. Bacterial artificial chromosome (BAC) clones (BACPAC Resources, Oakland, CA) were cultured, and BAC DNA was isolated using the BACMAX DNA isolation kit (Epicentre, Madison, WI). DNA was labeled with Spectrum Orange (Vysis, Downers Grove, IL) according to the manufacturer's instructions, precipitated, resuspended in hybridization buffer (Vysis), and hybridized to slides overnight at 37°C. Washed and dehydrated slides were mounted and counterstained with DAPI/antifade solution (Vysis) and visualized with an Olympus BX-61 fluorescent microscope equipped with a charge coupled device camera and Cytovision digital image acquisition system (Applied Imaging, San Jose, CA).

### Somatic cell hybrids

The proband's lymphoblasts were fused with thymidylate kinase-deficient RJK hamster cells and hybrid clones selected as described [13]. Colonies were selected in the presence of hypoxanthine-aminopterin-thymidine. After > 10 serial passages, DNA was extracted from clones and tested by PCR for chromosome 7 and 10. Positive clones were screened with distal 7p, 7q, 10p, and 10q microsatellite markers heterozygous in the proband, and a clone with the derivative 10 chromosome but not the derivative 7 chromosome or normal chromosome 7 or 10 was identified. Breakpoints were mapped by testing this hybrid clone for the presence or absence of chromosome 7 and 10 sequence tagged sites designed iteratively from the human genome sequence.

### Allelic expression

Intronic sequences were PCR amplified using primers CATTGCACGCTACGGAGTAA and TGCTTCACACACCTGCATCT (*MPP7*), TCCAAATCATTGTTTCTCAAACC and AATATTAGTTGGGCGTCGTG (*WAC*), and CCCACAACTGGCCTGTTAAA and CGAGGCCGGAAGTTAGTCTT (*MTERF*). PCR products from the proband's genomic DNA were sequenced and a heterozygous base identified in each gene. Heterogeneous nuclear RNA was isolated from the patient's transformed lymphoblasts by repeated treatments with NP-40 lysis buffer (10 mM Tris pH 4.0, 10 mM NaCl, 3 mM MgCl_2_, 0.5% NP40) followed by centrifugation. The nuclear pellet was resuspended in Tripure (Roche, Indianapolis, Indiana), and the iScript cDNA synthesis kit (BioRad, Hercules, California) was used to make cDNA. Reverse transcriptase PCR (RT-PCR) products were amplified from DNAse-treated cDNA isolated from lymphoblastoid cells carrying the balanced translocation and sequenced as described above. Control reactions omitting reverse transcriptase were performed to rule out amplification of contaminating genomic DNA. Electropherograms of genomic versus cDNA sequences were compared to determine whether both alleles of *MPP7*, *WAC*, and *MTERF *were expressed.

## Results

Coding sequences of the genes causing MODY1-4 and MODY6 (*GCK*, *HNF1A*, *IPF1*, *NEUROD1*, and *HNF4A*) were sequenced in the proband to rule out known causes of MODY with compatible clinical presentations. No mutations were found in any of these genes. The *HNF1B *gene causing MODY5 was not sequenced because of the distinct clinical presentation of this form of MODY. Two candidate genes in the cytogenetic vicinity of the 10p12 translocation breakpoint, *PTF1A *and *GAD2 (GAD65)*, were also investigated by FISH. Neither gene was deleted or disrupted by the translocation (data not shown). High resolution oligonucleotide array CGH did not reveal any cryptic duplications or deletions near the translocation breakpoints or any pathologic copy number variation elsewhere in the genome (data not shown).

**Figure 2:**
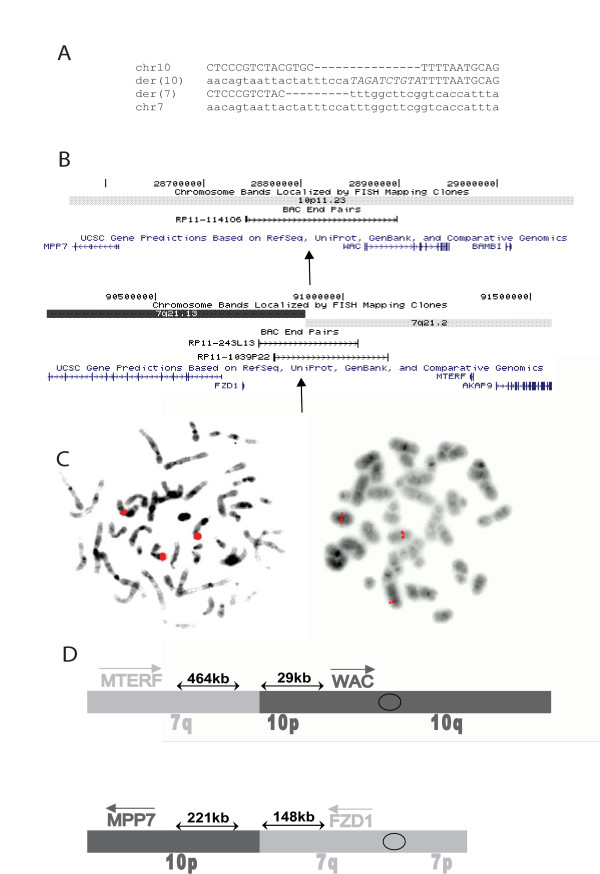
**A. Sequences of PCR products from junctions aligned to reference human genome sequence**. Upper case, chromosome 10 sequences. Lower case, chromosome 7 sequences. Upper case italics, origin unknown. B. UCSC Genome Browser tracks showing genes flanking the translocation breakpoints (arrows) and BAC clones used for FISH. **C**. FISH showing three signals for chromosome 7 (left) and chromosome 10 (right) BAC probes shown in B. **D**. Cartoon illustrating orientation of flanking genes and relative distances from translocation breakpoints.

We performed additional FISH studies using BAC clones from chromosomes 7 and 10 to narrow the location of the breakpoints. Concurrently, we generated somatic cell hybrids of the proband's lymphocytes and hamster cells. We obtained one hybrid clone containing the derivative 10 chromosome but not the normal chromosome 10 or the derivative 7 chromosome. We then used this hybrid clone to map the breakpoints by sequence tagged site content mapping, using sequences near the FISH-delineated breakpoints. Ultimately we identified sequences sufficiently close to both breakpoints to design PCRs that amplified both junction fragments. Alignment of the junction sequences to the reference human genome sequence revealed that the breakpoints were at chr10:28,832,302 with a four nucleotide deletion (chr10:28,832,303–28,832,306) on the derivative 7 chromosome and at chr7:90,883,582 with a ten nucleotide insertion of *TAGATCTGTA *on the derivative 10 chromosome (Fig. 2A).

To confirm these breakpoints we performed FISH on the subject's immortalized lymphoblasts using BAC clones RP11-1141O6 (chromosome 10) and a combination of RP11-243L13 and RP11-1039P22 (chromosome 7 breakpoint) (Fig. 2B). Both hybridizations showed three signals, as expected (Fig. 2C). Although RP11-243L13 was predicted to cross the chromosome 7 breakpoint determined by PCR, this BAC clone gave only two signals, probably because of the abundance on one side of the breakpoint of repetitive sequences whose hybridization was suppressed by Cot1 DNA. G-banded karyotyping confirmed that the metaphase cells used for FISH had the same karyotype as the proband's peripheral blood cells.

**Figure 3:**
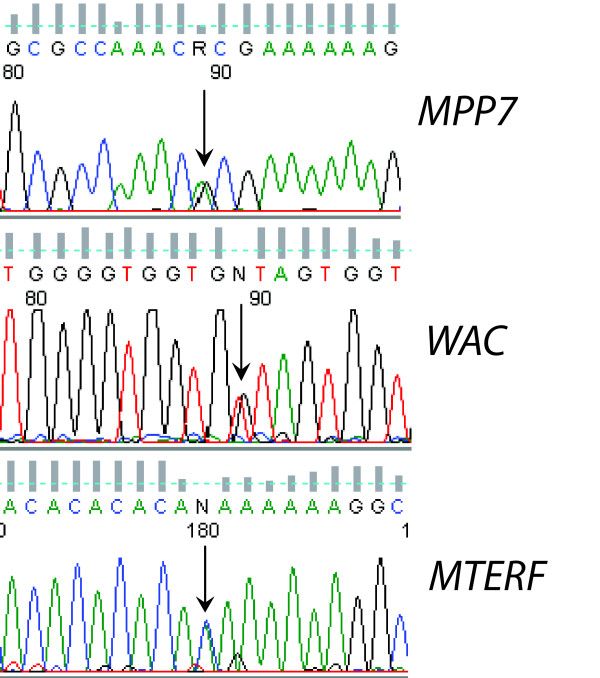
**Electropherograms showing heterozygous nucleotides (arrows) in RT-PCR products from *MPP7*, *WAC*, and *MTERF *heterogeneous nuclear RNA indicating biallelic transcription**.

Neither breakpoint directly disrupted any known protein coding gene, microRNA, or other annotated functional genomic element (Fig. 2B). We therefore investigated genes neighboring the breakpoints that could be subject to position effects. No genes within 1 megabase (Mb) were known to play a role in pancreatic islet cell function. To examine whether the translocation affected expression of nearby genes, we attempted to identify expressed polymorphisms in the four genes closest to the breakpoints (Fig. 2D). We sequenced exons but did not find any heterozygous variations in either coding or untranslated regions in these genes. We then identified heterozygous intronic SNPs in *MPP7*, *WAC*, and *MTERF *that could be used to examine allelic expression in heterogeneous nuclear RNA, as described [24]. *FZD1 *is intronless and thus could not be assayed in this fashion. *MPP7*, *WAC*, and *MTERF *all showed biallelic transcription, with approximately equal abundance of the two alleles (Fig. 3). Thus there did not appear to be any major position effects on expression of genes flanking the translocation breakpoints in lymphoblastoid cells.

## Discussion

MODY is a monogenic form of diabetes mellitus. We hypothesized that MODY-like diabetes in the proband resulted from disruption of a gene by his balanced translocation. Our proband did not meet the strict diagnostic criteria for MODY. His age at diagnosis was 32 years, and there was no clear pattern of autosomal dominant inheritance. The lack of inheritance would be expected if the translocation caused MODY and occurred *de novo*. The mother was diabetic, but the clinical features and course of her disease were consistent with type 2 diabetes rather than MODY. Since our patient has an insulin secretion defect, no evidence of insulin resistance, and an indolent disease course, we speculate that he and his mother have distinct etiologies of their diabetes. The daughter who carries the translocation was not diabetic at the time of this study, but it is possible she will develop diabetes later in life.

One cytogenetic breakpoint was in band 10p12. At least two genes in this region have been implicated in pancreatic development and physiology. *PTF1A *has previously been shown to play a role in islet cell development. *GAD2 *(also called *GAD65*) encodes glutamate decarboxylase 2, is a target for islet cell antibodies in type I diabetes [25]. FISH studies showed that neither gene was disrupted or deleted by the translocation. We were ultimately able to show that the chromosome 10 breakpoint was at least 5 Mb away from either gene's coding sequence, with multiple intervening genes. This distance is much greater than the maximum distance (~1 Mb) over which position effects have been described for other diseases due to balanced translocations [26]. No other genes in the immediate vicinity of the translocation breakpoints have been implicated as diabetes candidate genes in genome-wide association studies [3,27,28].

Sequencing the junctions revealed that the translocation was for all intents and purposes molecularly balanced, with ten nucleotides inserted at the derivative 10 junction and four nucleotides deleted from chromosome 7. Neither breakpoint lay in any apparent repeated sequence motif, and the origin of the ten nucleotide sequence inserted at the derivative 10 junction is unknown. Neither breakpoint disrupted any annotated gene, and there were no genes previously implicated in pancreatic islet cell function within 1 Mb of either breakpoint. We examined the allelic expression of genes flanking the breakpoints in lymphoblastoid cells. While we found no evidence of abnormal expression in these cells, we cannot exclude tissue-specific effects on gene expression and/or fusion transcripts. For instance, campomelic dysplasia is clearly the result of mutations or balanced translocations altering expression of the *SOX9 *gene, but in one case resulting from a balanced translocation, there was no difference in transcription level between the two *SOX9 *alleles in lymphoblasts [29].

One gene 221 kilobases downstream from the 10p breakpoint, *Palmitoylated Membrane Protein 7 *(*MPP7*), merits further consideration as a candidate diabetes gene. According to the Novartis Gene Expression Atlas [30], mouse *Mpp7 *is expressed broadly but most highly in pancreas. In this same database, human *MPP7 *is expressed in pancreatic islets. *MPP7 *encodes a member of the membrane-associated guanylate kinase (MAGUK) family [31]. MAGUK proteins are found at areas of cell-cell contact, where they are essential for multi-protein complex assembly. MPP7 forms a tripartite complex with Discs Large 1(DLG1) and Lin7, and is necessary for maintenance of cell polarity [32,33]. Interestingly, *HNF4A*, the gene responsible for MODY1, has also been shown to be important in formation of tight junctions [34]. *HNF4A *overexpression in embryonal carcinoma cells causes the formation of tight junctions in a dose-dependent manner [34,35]. Tight junction-associated proteins are upregulated in islets during maturation and may be necessary in mature beta cells for proper glucose-stimulated insulin secretion [36-38]. Furthermore, glucose upregulates tight junctions in a dose-dependent manner in cultured rat islets [39]. It has been theorized that tight junctions are essential to separate the high concentrations of glucagon, insulin, and somatostatin in the apical surface from their receptors on the basal surface, which could pathologically inhibit secretion via autoregulation [40-42]. Thus altered expression of *MPP7 *in islets might affect cell polarity and impair glucose-stimulated insulin secretion, resulting in diabetes.

## Conclusion

We mapped the breakpoints of an apparently balanced 7;10 translocation associated with MODY-like diabetes to nucleotide resolution. The translocation and diabetes in the proband could be coincidental: apparently balanced non-Robertsonian translocations have been found in about 1 in 1400 consecutive newborns [43]. No gene was obviously disrupted by the translocation, but the chromosome 10 breakpoint was near *MPP7*, a plausible biological candidate gene for diabetes by virtue of its function in cell polarity. Screening of additional diabetic subjects for *MPP7 *mutations and generation of *Mpp7 *knockout mice are needed to test the hypothesis that this gene, and by inference abnormal islet cell polarity, play a role in impaired glucose-stimulated insulin secretion in MODY or other forms of diabetes mellitus.

## Abbreviations

CGH: comparative genomic hybridisation; BAC: Bacterial artificial chromosome; BMI: Body mass index; *DLG1*: *Discs large 1*; DAPI: 4',6-diamidino-2-phenylindole; FISH: Fluorescent in-situ hybridisation; *FZD1*: *Frizzled 1*; *GAD2*: *Glutamic Acid Decarboxylase 2*; *GAD65*: *Glutamic Acid Decarboxylase 65*; *GCK*: *Glucokinase*; *GLUT2*: *Glucose transporter 2*; *HNF1A*: *Hepatocyte nuclear factor 1 alpha*; *HNF1B*: *Hepatocyte nuclear 1 beta*; *HNF4A*: *Hepatocyte nuclear factor 4 alpha*; *HOMA*: *Homeostatic model assessment*; *IGF2*: *Insulin-like growth factor 2*; *IPF1*: *Insulin promoter factor 1*; *IHPK1*: *Inositol hexaphosphate kinase 1*; MAGUK: Membrane-associated guanylate kinase Mb; Megabase; MODY: Maturity-onset diabetes of the young; *MPP7*: *Membrane protein, palmitoylated 7*; *MTERF*: *Mitochondrial transcription termination factor*; *NEUROD1*: *Neurogenic differentiation factor 1*; *PTF1A*: Pancreas specific transcription factor 1a; RT-PCR: Reverse transcriptase polymerase chain reaction; SNP: Single nucleotide polymorphism; *SOX9*: *SRY-box containing gene 9*; STS: Sequence-tagged site; *WAC*: WW domain-containing adapter with a coiled-coil.

## Competing interests

The authors declare that they have no competing interests.

## Authors' contributions

EJB performed molecular cytogenetic and genetic analyses and drafted the manuscript. SR and MGB identified the proband and characterized his diabetes. GB helped perform molecular cytogenetic studies. RAS helped plan and interpret conventional and molecular cytogenetic studies. ARZ conceived of the study, helped interpret data, and revised the manuscript.
